# Reproducibility of the Optical Biometer OA-1000 (Tomey)

**DOI:** 10.1155/2014/814761

**Published:** 2014-04-10

**Authors:** Susanne Christiane Goebels, Berthold Seitz, Achim Langenbucher

**Affiliations:** ^1^Saarland University Medical Center, Kirrberger Strasse 100, Building 22, 66421 Homburg/Saar, Germany; ^2^Experimental Ophthalmology, Saarland University, Kirrberger Strasse 100, Building 22, 66421 Homburg/Saar, Germany

## Abstract

*Aim*. The OA-1000 (Tomey, Japan) is a new optical biometer, which measures axial length (AL), anterior chamber depth (ACD), and central corneal thickness (CT) utilizing optical interference technology. The aim of this study was to prove the reproducibility which is considered fundamental for other clinical investigations. *Methods*. 55 healthy volunteers were enrolled in this study. For each measurement of AL, ACD, and CT the biometer is grabbing a sequence of 10 shots and mean value (mean) and standard deviation (SD) are displayed. Five consecutive measurements were performed and average and standard deviation were assessed. Cronbach's α was derived as a quality measure for reproducibility. *Results*. For AL measurement Cronbach's α was 1.000, for CT 0.999, and for ACD 0.979, respectively. Mean value for AL was 23.36 ± 1.03 mm, for ACD it was 3.60 ± 0.687 mm, and for CT it was 552.08 ± 29.70 **μ**m, respectively. Standard deviation for AL was 0.013 ± 0.022 mm, for ACD 0.09 ± 0.11 mm, and for CT 2.18 ± 1.75 **μ**m. One correlation was found between mean values for AL and ACD (*R* = 0.388, *P* = 0.005); no other correlations were found between mean values or values of standard deviation of AL, ACD, or CT. *Conclusion*. The OA-1000 shows an excellent reproducibility for measurement of AL, ACD, and CT and can be used in clinical practice.

## 1. Background and Purpose

With the improvement of surgical techniques, for example, in cataract and refractive surgery, optimized calculation schemes, and improved quality of intraocular implants, the demands on accurate biometric data have been increased significantly in the last decade.

In general, accurate biometry is crucial for reaching the goal of intended postoperative refractive outcome. Based on classical schematic model eyes such as the Gullstrand eye, a measurement error of 1 mm in AL ends up in an error refraction of around 3.5 D and an erroneous determination of the pseudophakic lens position of 1 mm based on the preoperative assessed phakic ACD leads to an error refraction of around 1.6 D. Norrby found in 2008 a slightly lower effect and provided an error of refraction of 2.8 D with an AL difference of 1 mm [1]. Especially for newer generation lens implants such as multifocal (either refractive or diffractive) or accommodating intraocular lens implants an inappropriate measurement of axial length or anterior chamber depth could cause significant deterioration of visual performance after cataract surgery [2] or even a loss of functionality (e.g., near and far distance vision in multifocal lenses).

Since 1999 the IOLMaster (Carl Zeiss Meditec AG, Germany) has been available and is nowadays commonly used in clinical practice [3]. ACD is measured using slit projection technique, which is known to be limited in precision and does not properly work in pseudophakic eyes. Therefore, new optical biometers have been developed for measuring phakic and pseudophakic ACD, which are working according to the optical coherence interferometry principle such as the LenStar (Haag Streit, Switzerland), AL-Scan (Nidek, Japan), or the OA-1000 (Tomey, Japan).

The OA-1000 (Tomey, Japan) is an optical biometer which measures the optical distance between ocular surfaces utilizing optical interference technology. This device is designed to measure axial length (AL), anterior chamber depth (ACD), and central corneal thickness (CT) [4].

The OA-1000 is based on light interference, which measures in three steps different anatomical structures using a super luminescent diode (SLD) within a wavelength range from 820 to 850 nm. The measurement range for the AL is reported to be 14–40 mm, for ACD it is 1.5–7.0 mm, and for corneal thickness it is 200–1200 *μ*m [4]. The precorneal tear film, the corneal back surface, and the front surface of the (phakic) lens as well as the retinal pigment epithelium are extracted from the waveform based on signal processing techniques.

The OA-1000 offers the advantages of a noncontact method that requires only minimal training for the examiner, no error induced by manual alignment due to a highly efficient autoshot mode, and short measurement time as outlined in a previous paper [5].

The goal of the present study was to evaluate the reproducibility of the new optical biometer OA-1000 in a clinical setup based on measurements of healthy patients.

## 2. Subjects and Methods

Between May and June 2012 we examined 55 healthy volunteers from the staff of the Department of Ophthalmology at the Saarland University Medical Center. The volunteers were fully informed about the purpose of this study and the study was performed according to the Declaration of Helsinki. We examined 45 female subjects (81.8%) and 31 right eyes (56.3%). Mean age was 38.8 ± 12.5 years (range from 18 to 64 years).

**Figure 1:**
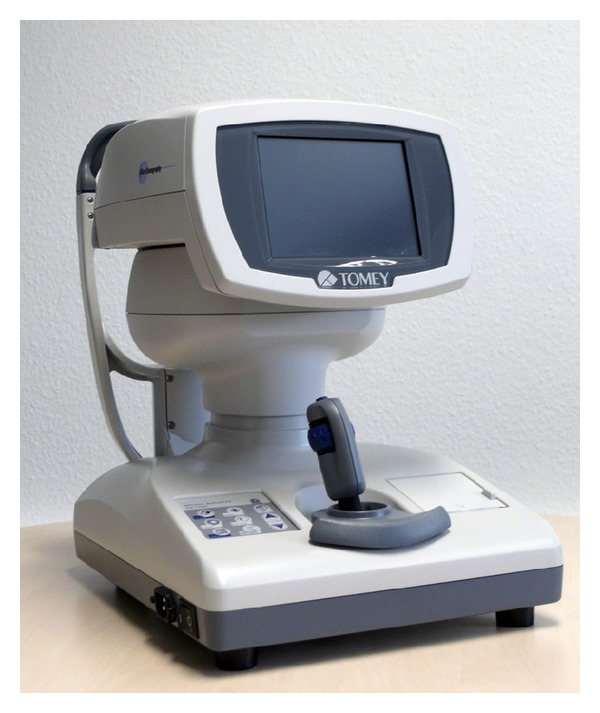
The OA-1000 by Tomey is a new optical biometer designed to measure axial length, anterior chamber depth, and corneal thickness.

**Figure 2:**
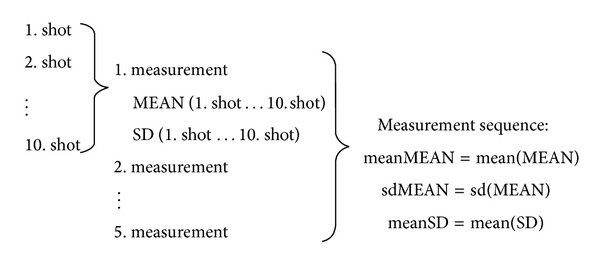
Strategy of measurements with the OA-1000. A sequence of 5 measurements was taken and each measurement consisted of 10 shots.

With the optical biometer OA-1000 (Figure 1) we measured axial length, (external) anterior chamber depth, and corneal thickness. Each measurement consists of 10 shots and the mean value (MEAN) and standard deviation (SD) were displayed automatically at the end of the measurement for all three measurement parameters (Figure 2).

Five repeated measurements were performed in a sequence by one examiner (G. S.) in one eye of each volunteer to evaluate the reproducibility of the OA-1000. It was ensured that after each measurement the head of the volunteer was newly positioned on the chin rest and the biometer was realigned. We evaluated MEAN and SD of all measurements.

The average value of the 5 mean values for each eye (meanMEAN) for AL, ACD, and CT was recorded to display an overall measure of our patient dataset. The standard deviation of mean values (sdMEAN) for AL, ACD, and CT was recorded to give an insight about the variation of the 5 measurements in each measurement sequence and the robustness of the measurement output. The average of the SD values (meanSD) for AL, ACD, and CT was derived to condense the standard deviations of all 5 measurements of a measurement sequence. For evaluation of potential correlations between AL, ACD, and CT we determined Pearson's rank correlation coefficient. *P* values less than 0.05 were considered statistically significant. Cronbach's *α* was determined for the 5 repeated measurements in measurement sequence. Cronbach's *α* is a measure of reliability. More specifically, *α* is a lower bound for the true reliability of the survey. The computation of Cronbach's *α* is based on the number of items on the survey (k) and the ratio of the average interitem covariance to the average item variance. The simplifications which are made in the mathematical formalism of deriving Cronbach's *α* are based on the assumption that the item variances do not differ between the 5 repeated measurements (5 measurements) in our measurement sequence, which has been proven with the standard deviation of the mean values (sdMEAN) and average of standard deviations (meanSD) for all 3 measurement parameters. In a clinical environment, *α* values of 0.9 or higher indicate a good reliability (or repeatability) of the output values.

Statistical analysis was performed using SPSS commercial statistical analysis software program (SPSS version 19.0, IBM, Chicago, USA).

## 3. Results

### 3.1. Descriptive Statistics

Average of all mean of five repeated measurements (meanMEAN) for all 55 individuals for AL was 23.36 mm (range from 20.66 mm to 26.11 mm), for ACD it was 3.67 mm (range from 2.5 mm to 5.32 mm), and for CT it was 552.08 *μ*m (range from 485.20 *μ*m to 663.6 *μ*m).

Average of all standard deviations of five repeated measurements (sdMEAN) for AL was 0.08 mm (range from 0.0 mm to 0.15 mm), for ACD it was 0.090 mm (range from 0.0 mm to 0.44 mm), and for CT it was 2.18 *μ*m (range from 0.55 *μ*m to 8.82 *μ*m). In Table 1 the average values and standard deviations of these parameters are listed.

**Figure 3:**
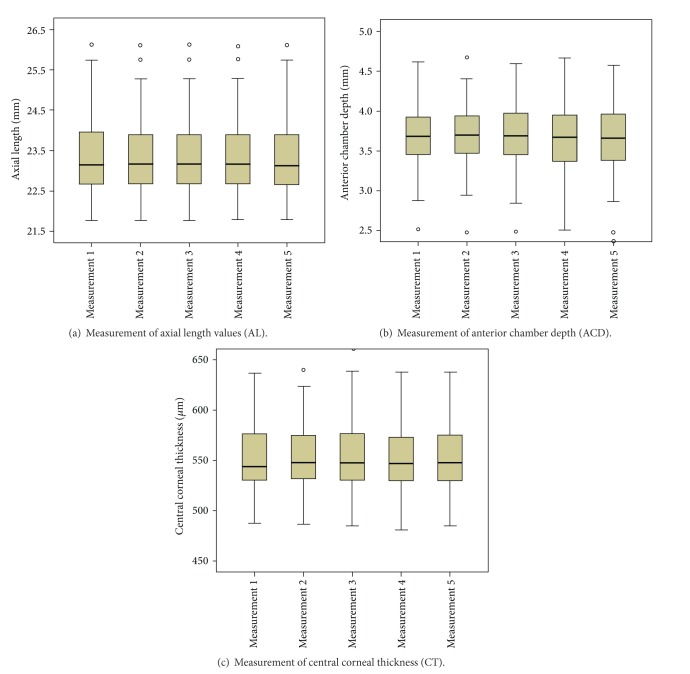
Boxplots show for all 5 measurements in the measurement sequence the median, 25% and 75% quartile, as well as the 95% confidence interval of measurements. None of the measurement parameters show a drift from the first to last measurement.

Average of all standard deviations of five repeated measurements (meanSD) for AL was 0.008 mm (relative variation 0.0325%), for ACD it was 0.02 mm (relative variation 0.5471%), and for CT it was 4.11 *μ*m (relative variation 0.735%). Average values and standard deviations of these parameters are listed also in Table 1. In Figure 3 the median, 25% and 75% quartile, as well as the 95% confidence interval for all 5 measurements in the measurement sequence is shown.

### 3.2. Correlations and Reliability of the Biometer

Only one correlation was found between meanMEAN of axial length and meanMEAN of anterior chamber depth (*R* = 0.388, *P* = 0.005). Excepted this no further correlations were found between the mean values (meanMEAN) of AL, ACD, or CT nor between standard deviations of mean values (sdMEAN) of AL, ACD, and CT; the results are listed in Table 2. Cronbach's *α* for AL was 1.000, for CT it was 0.999, and for ACD it was 0.979, respectively.

## 4. Discussion

In modern cataract and refractive surgery accurate measurements of ocular dimensions are getting more and more important. Accurate measurements of AL, ACD, and keratometry or corneal topography are essential parameters for the calculation of the appropriate IOL power and for getting optimal postoperative results in cataract surgery [6]. A dissatisfied patient due to unexpected postoperative refraction error is the major reason for IOL explantation [6, 7].

In a previous study we compared the OA-1000 results of axial length and anterior depth with those obtained with the IOLMaster and contact applanation A-scan ultrasonography. We found differences, especially in measuring anterior chamber depth, but all results correlated well with the values of IOLMaster and AL-3000 [5]. But a crucial step in assessment of a new instrument is in general the proof of consistency of the measurement results, which is the task of the present study.

First, the standard deviation between the 10 shots of a single measurement of axial length, anterior chamber depth, or central corneal thickness is very small in a normal population using the OA-1000. The measured mean standard deviation of 8 *μ*m in AL provides an error of refraction of 0.02 D in IOL power calculation [1]. In addition, the variation of axial length, anterior chamber depth, and corneal thickness measurements in repeated measurements of an individual is very small, which indicates an excellent reproducibility of the measurement values. There are no significant differences comparing mean standard deviation measurements from 1 to 5, which indicate that there is no systematic drift. Furthermore, it verifies that a single measurement is representative and sufficient as an estimate for this measure. A correlation was found between the mean value of ACD and the mean value of AL, which implies that in larger eyes (myopic eyes) the anterior chamber depth is larger compared to shorter (hyperopic) eyes. But this fact is already well experienced. All other variables do not show significant cross-correlations, which indicates that there are no trend errors which could falsify the measurement results.

We found excellent values for reproducibility for AL, ACD, and CT. In a ranking, the reliability of axial length was best followed by central corneal thickness and anterior chamber depth. One major reason for a lower value for reproducibility in measuring ACD compared to AL and CT could be that all individuals included in the present study were young and healthy normals with a sufficient physiological accommodation, which is known to affect the anterior chamber depth [8]. This effect could be avoided using topic cycloplegic eye drops during measurements with the side effect of losing the privilege of a noninvasive measurement technique. A second reason could be that the ACD measurement has to be performed along the “symmetry axis” of the eye, which ensures that the measurement is perpendicular to the corneal and lens front surface and therefore we get a sufficient signal-to-noise ratio. To avoid measuring along the “visual axis,” which accord to the line of sight and end up in the fovea and not in the posterior geometric pole, the fixation target of the device has to be moved out of the measurement axis manually. This could be performed in discrete steps. This slight axis shift between the measurement axis and the line of sight has to be dealt carefully with and inappropriate shift results in incorrect ACD values due to an oblique measurement through the anterior chamber of the eye. The lower reproducibility of central corneal thickness in comparison to axial length values might be due to tear film irregularities.

Concerning reproducibility other optical biometers lead to similar results. The LenStar (Haag-Streit AG, Koeniz, Switzerland) is another new but already evaluated biometer. Rohrer et al. tested the reliability of the LenStar. Similar values are found for standard deviation in the test setup of repeated measurements. The mean/standard deviation of standard deviation of axial length measurement was 0.025/0.026 mm, for ACD it was (0.02/0.03 mm and therefore slightly better with the OA-1000, and for CT it was slightly better with the LenStar (2.2/2.0 *μ*m) [6]. Buckhurst et al. studied the reproducibility of the LenStar and referenced to the respective values derived with the IOLMaster and ultrasound biometer [9]. They found a good repeatability (≤2% of the average value of each biometric measurement) for the LenStar. Average standard deviation for AL was 16 *μ*m in one session and 6 *μ*m between sessions, for ACD it was 0.051 mm and 0.013 mm, and for CT it was 0.003 mm and 0.001 mm. Hildebrandt et al. described a good reproducibility for the LenStar in pseudophakic eyes with a standard deviation of 0.02 mm for AL measurement [10]. Holzer et al. confirmed high precision of the LenStar in healthy eyes [3]. Cruysberg et al. reported reproducibility better than 0.9% for the LenStar [11].

Limitations of our study were that we measured only healthy volunteers of our Department of Ophthalmology with a small range of age range between 18 and 64 years. Our results do not fully reflect the conditions of an elderly (cataractous) or nonhealthy study population. We did not use cycloplegic medication; therefore, physiological accommodation was not excluded.

One disadvantage of OA-1000 is that it is not measuring corneal curvature. Therefore, a keratometer or corneal topographer is required to collect all values which are needed for IOL power calculation. In contrast, the IOLMaster or LenStar is able to measure both optical distances in the eye and corneal curvature. But especially in the last decade the customization of intraocular lens implants progressed a lot and lenses are individualized for compensating spherical aberration of the eye or correcting wavefront errors. To calculate those customized lens implants properly standard keratometry as it is integrated in the IOLMaster or in the LenStar is not fully sufficient and a (high resolution) corneal topography of the anterior and posterior corneal surface is required to derive the appropriate intraocular lens implant, for example, based on numerical ray tracing techniques.

Further, the LenStar is measuring all values alongside the visual axis, whereas with the IOLMaster as well as the OA-1000 the optical pathway has to be changed if switching from axial length measurement to anterior chamber measurement [6]. The advantage of the OA-1000 in comparison with the IOLMaster might be the reliable measurement of ACD in phakic and pseudophakic eyes, even if we did not test the reliability of ACD measurement in pseudophakic eyes in the present study. The IOLMaster, which is the gold standard in biometry today, allows precise and reliable measurement of AL, but is known to be less accurate in measuring ACD due to slit projection techniques. In assessing the ACD with IOLMaster, a slit beam illumination at an angle of 30° is used which leads to lower resolution, reproducibility, and accuracy [6]. The internal ACD—the distance between corneal back surface and the front surface of the lens, cannot be measured directly but has to be derived based on the corneal radius using inverse ray tracing [6, 12].

## 5. Conclusion

In conclusion, the OA-1000 provides highly reliable results for axial length, anterior chamber depth, and central corneal thickness in healthy eyes. Its advantage is the excellent coherence of results in multiple measurements of axial length, anterior chamber depth, and central corneal thickness as well as the easy handling in an autoalignment mode. One has to be aware that a separate keratometry or topographic measurement is required to perform IOL calculation.

## Figures and Tables

**Table 1:** Average values of axial length, anterior chamber depth, and central corneal thickness. Mean/SD refers to the mean value/standard deviation of 10 shots within each measurement. Within a sequence of 5 repeated measurements, meanMEAN is the average of the MEAN, sdMEAN is the standard deviation of the MEAN, and meanSD is the average of the SD values. AL refers to axial length, ACD to anterior chamber depth, and CT to central corneal thickness.

	meanMEAN	sdMEAN	meanSD
AL (mm)	23.36 ± 1.032	0.076 ± 0.003	0.008 ± 0.003
ACD (mm)	3.673 ± 0.459	0.092 ± 0.119	0.02 ± 0.017
CT (µm)	552.08 ± 39.702	2.181 ± 1.750	4.106 ± 1.948

**Table 2:** Upper right section of the correlation matrix denotes significance levels, whereas the lower left denotes correlation coefficients according to Pearson's ranking correlation test.

		meanMEAN			sdMEAN		
		AL	ACD	CT	AL	ACD	CT
meanMEAN	AL		**0.388**	**0.132**	**0.012**	**−0.185**	**0.108**
	ACD	0.005		**−0.062**	**0.259**	**0.110**	**0.197**
	CT	0.337	0.668		**−0.109**	**0.045**	**0.019**

sdMEAN	AL	0.928	0.069	0.427		**−0.109**	**−0.070**
	ACD	0.210	0.445	0.758	0.452		**0.060**
	CT	0.432	0.170	0.889	0.613	0.679	
